# Harmonization of exosome isolation from culture supernatants for optimized proteomics analysis

**DOI:** 10.1371/journal.pone.0205496

**Published:** 2018-10-31

**Authors:** Agata Abramowicz, Lukasz Marczak, Anna Wojakowska, Szczepan Zapotoczny, Theresa L. Whiteside, Piotr Widlak, Monika Pietrowska

**Affiliations:** 1 Maria Sklodowska-Curie Institute - Oncology Center, Gliwice Branch, Gliwice, Poland; 2 Institute of Bioorganic Chemistry, Polish Academy of Sciences, Poznan, Poland; 3 Faculty of Chemistry, Jagiellonian University, Krakow, Poland; 4 Department of Pathology, Immunology and Otolaryngology, University of Pittsburgh School of Medicine and UPMC Hillman Cancer Center, Pittsburgh, Pennsylvania, United States of America; University of South Alabama Mitchell Cancer Institute, UNITED STATES

## Abstract

Exosomes, the smallest subset of extracellular vesicles (EVs), have recently attracted much attention in the scientific community. Their involvement in intercellular communication and molecular reprogramming of different cell types created a demand for a stringent characterization of the proteome which exosomes carry and deliver to recipient cells. Mass spectrometry (MS) has been extensively used for exosome protein profiling. Unfortunately, no standards have been established for exosome isolation and their preparation for MS, leading to accumulation of artefactual data. These include the presence of high-abundance exosome-contaminating serum proteins in culture media which mask low-abundance exosome-specific components, isolation methods that fail to yield “pure” vesicles or variability in protein solubilization protocols. There is an unmet need for the development of standards for exosome generation, harvesting, and isolation from cellular supernatants and for optimization of protein extraction methods before proteomics analysis by MS. In this communication, we illustrate the existing problems in this field and provide a set of recommendations that are expected to harmonize exosome processing for MS and provide the faithful picture of the proteomes carried by exosomes. The recommended workflow for effective and specific identification of proteins in exosomes released by the low number of cells involves culturing cells in medium with a reduced concentration of exosome-depleted serum, purification of exosomes by size-exclusion chromatography, a combination of different protein extraction method and removal of serum-derived proteins from the final dataset using an appropriate sample of cell-unexposed medium as a control. Application of this method allowed detection of >250 vesicle-specific proteins in exosomes from 10 mL of culture medium.

## Introduction

Exosomes are a subset of extracellular vesicles (EVs) with a diameter ranging from 30 to 150nm that originate from an endocytic compartment of parent cells. Exosomes are formed within multivesicular bodies (MVBs) via the process of inward invagination. MVBs contained intraluminal vesicles fuse with the plasma membrane of the components in a parent cell, releasing exosomes into extracellular space [1,2]. The topography of the exosome membrane components and the content of their lumen resemble those of the parent cell [3]. This biogenesis process accounts for the importance of exosomes freely circulating in all body fluids as potential surrogates of parent cells and as a “liquid biopsy” of the tissue containing these parent cells. Recent interest in establishing exosome molecular profiles has led to an explosion of methods for their isolation from supernatants of cells and/or various body fluids (reviewed by Alvarez et al. [4] and Kalra et al. [5]). However, despite numerous methodologies introduced for exosome isolation, no single standardized and validated method has emerged to date; the issue is discussed in a review paper by Abramowicz et al. [6]. The nomenclature for EVs remains undefined, and exosome identity or their distinction from other, larger EVs remains unclear. Separation of exosomes from “contaminating” proteins present in cellular supernatants or body fluids used as sources of exosomes represents a major challenge because camouflaging of low-abundant proteins by high-abundant proteins is a common problem in mass spectrometry (MS). Another barrier to the characterization of isolated exosomes is their poor or incomplete recovery due to vesicle aggregation or a loss of exosomes during isolation procedures. These and other pitfalls of exosome isolation and proteomics workflows might contribute to the biased interpretation of results, incomplete definition of exosome cargos or, in the worst case, to a mistaken recognition of contaminants as *bona fide* components of exosomes [7,8].

The goal of this study was to identify and eliminate the “trouble spots” that often arise during preparation of exosomes for the MS analysis. The multi-step process of sample collection, exosome isolation, and extraction of exosome proteins for the subsequent MS analysis requires special attention to reduce the risk of introducing experimental artifacts. These may include cell culture media-derived contaminants, biofluid components masquerading as components of the exosome cargo or protein losses occurring during exosome extraction for MS analysis. Here, we consider steps that can be taken to harmonize experimental conditions to improve the quality of proteomic analysis of exosomes and provide recommendations for optimization of the detection of low abundance proteins that are true components of exosomes rather than contaminating serum or plasma artifacts.

## Results

### Removal of fetal bovine serum from cell culture supernatants to be used for exosome isolation

Cell culture media used for exosome harvest and isolation are usually supplemented with fetal bovine serum (FBS). High abundant serum proteins such as bovine albumin and bovine EVs are potential sources of “contaminants” and can lead to detection errors in subsequent MS analyses. When the amino acid sequence of the 20 most commonly identified exosome proteins deposited in the ExoCarta database [9] was analyzed (S1 Table), a high degree of similarity (often >95%) between homologous proteins in human and bovine exosomes was revealed. In fact, some proteins, including actin, elongation factor 1-α-1 or 14-3-3 protein zeta/delta were indistinguishable in the two species, implying that some bovine and human proteins cannot be differentiated by bioinformatics analysis of MS data. Indeed, when the raw MS/MS data registered for the exosome fraction were analyzed with the Mascot search engine using either human or bovine protein databases, the analysis returned 131 proteins that were specifically of bovine origin (i.e., putative components of FBS) and 170 proteins that were specifically human (i.e., putative exosome components). However, there were 135 proteins identified in both the bovine and human databases. These data showed that very high levels of homology of human and bovine proteins exist and comprise a large set of potential exosome components (S1 Fig).

**Fig 1:**
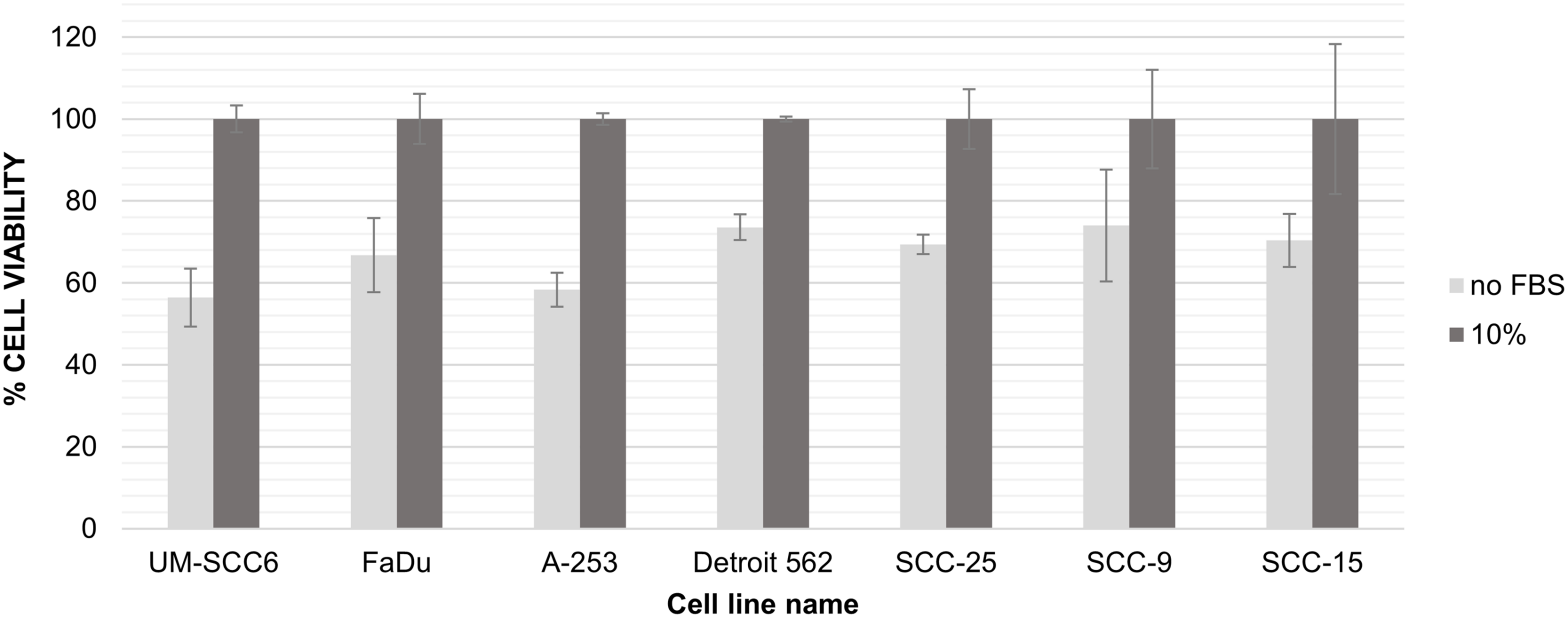
Effects of fetal bovine serum (FBS) on cell viability. The cell viability assessed for the panel of head and neck cancer cell lines cultured for 24 h in a medium with 10% FBS or without FBS supplementation.

The absence of FBS in cell culture media would be expected to solve the above-described problem of bovine artifacts. However, serum deprivation can markedly affect cell growth and viability. To determine whether removal of FBS for the final 24 h of culture would decrease the presence of bovine proteins in exosome-rich supernatants, seven different human head and neck cancer (HNC) cell lines were cultured without FBS during the last 24 h of incubation. As shown in Fig 1, this 24h-deprivation of FBS resulted in significant inhibition (approximately 33%) of viability in all seven cell lines, with UM-SCC6 and A-253 showing the greatest growth suppression that exceeded 40% relative to the “standard” FBS-supplemented medium.

**Fig 2:**
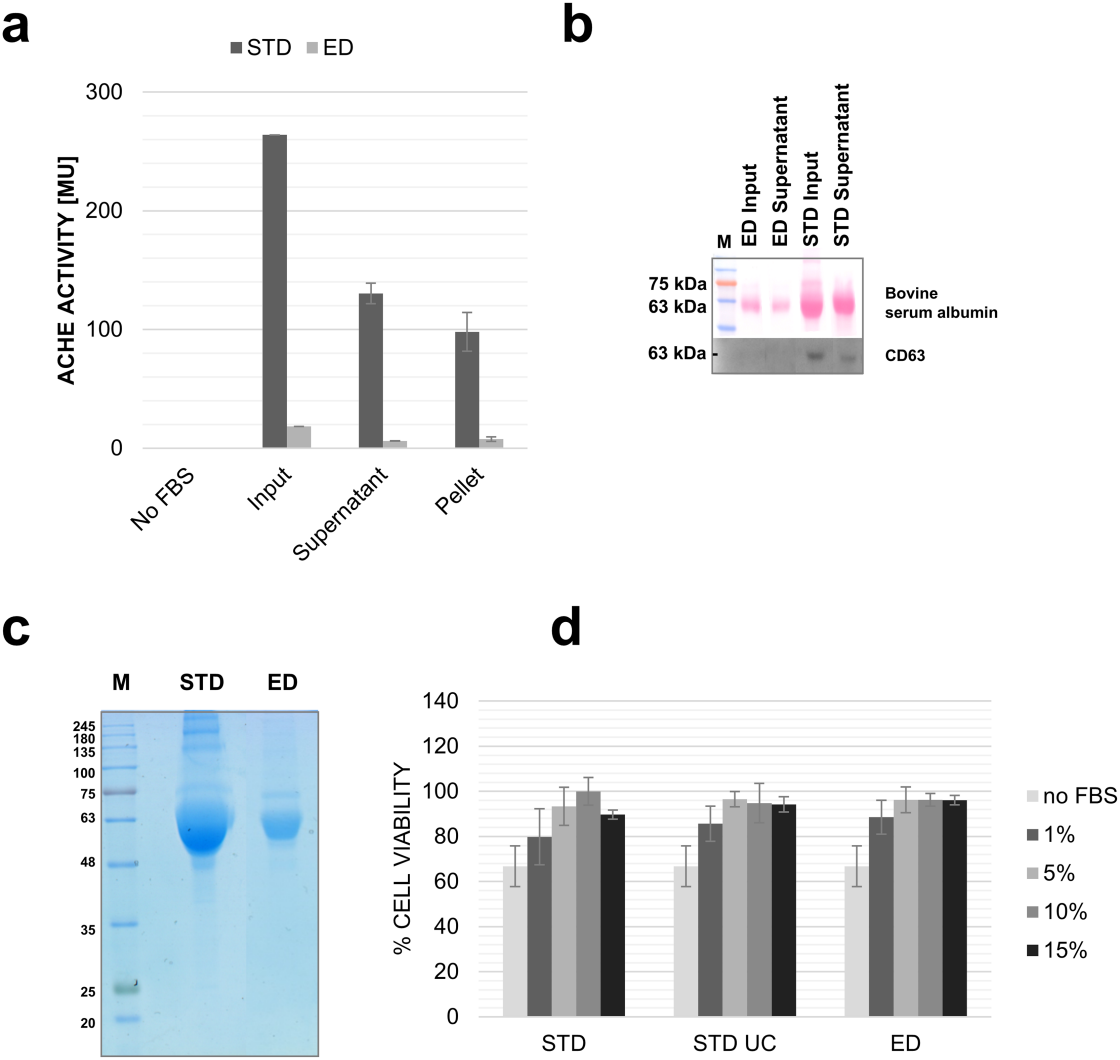
Characteristics of exosome-depleted FBS. In (a) Quantification of exosomes present in the cell culture medium supplemented with STD FBS or ED FBS at the input and in the post-UC fractions (supernatant and pellet) based on ACHE activity. In (b) Western blot of CD63 in the cell culture medium either before (input) or after UC (supernatant); Ponceau S staining demonstrates the serum albumin content. In (c) Coomassie blue staining of electrophoretically-separated proteins from standard (STD) and exosome-depleted (ED) FBS. In (d) Effects of supplementation with FBS on cell viability; FaDu cells were cultured for 24 h in a medium supplemented with STD FBS, ultracentrifuged STD FBS (STD-UC) or ED FBS.

The widely used alternative is to use media supplemented with FBS depleted of bovine exosomes by (a) overnight ultracentrifugation (UC) of medium containing FBS at 100,000 × g [10] or (b) by the use commercial exosome-depleted (ED) FBS. We compared the two approaches for efficacy in eliminating bovine contaminants from media supplemented with 20% (v/v) standard (STD) FBS or commercial ED FBS; each FBS was used after UC or without UC. Acetylcholinesterase (ACHE) activity and Western blot detection for CD63 were used to detect the presence of bovine exosomes. Fig 2a shows that UC of STD media decreased ACHE activity and CD63 marker by 50% only. On the other hand, ACHE activity and CD63 protein were barely detectable in the ED medium either pre- or post-UC (Fig 2b), indicating that the “contamination” with bovine exosomes could be substantially decreased using cell cultures with ED FBS but not with STD FBS depleted of exosomes by UC. Moreover, Fig 2c shows that ED FBS had a substantially lower protein content than STD FBS by Coomassie blue staining. When cells cultured in media supplemented with ED FBS and STD FBS were compared, no significant difference in growth of FaDu cells was detected (Fig 2d). The ED FBS content as low as 1% in the medium did not significantly impair cell growth while decreasing the content of bovine proteins in culture supernatants. However, culturing of cells in media supplemented with 1% FBS impaired viability of certain cell types in longer experiments (48–72 h; data not showed). Hence, FBS concentration lower than 5% could not be recommended as a general solution.

### Exosome isolation from supernatants of tumor cell cultures by size exclusion chromatography

**Fig 3:**
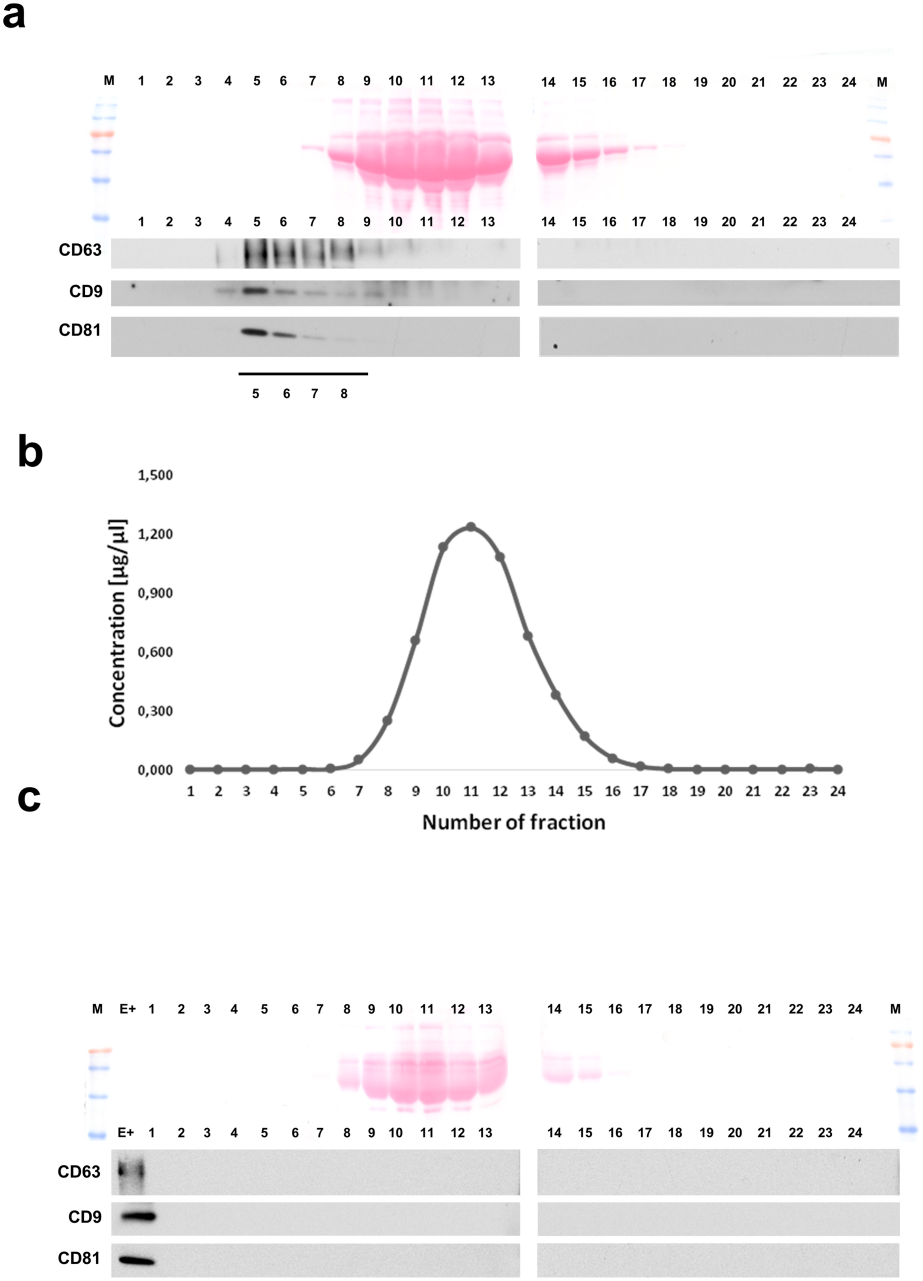
Exosome isolation by the size exclusion chromatography. In (a) representative immunoblot showing the distribution of exosome markers (CD63, CD9, CD81) and high-abundance serum proteins (illustrated by Ponceau S staining) in the successive SEC fractions of a FaDU culture medium. In (b) total protein concentrations (μg/uL) in the subsequent SEC fractions. In (c) culture medium supplemented with 5% ED FBS and NOT co-cultured with cells was analyzed as in Panel A; “E+” denotes exosome-containing positive control.

Exosomes released by FaDu cells were isolated from cell culture supernatants by size exclusion chromatography (SEC). Fig 3a shows that exosomes, monitored by Western blots as CD63+, CD9+ or CD81+ vesicles, were eluted in fractions # 5–8 with a gradual decrease in the later fractions. In the complementary Ponceau stain of proteins on nitrocellulose membranes, the most intensive bands appeared in fractions # 8–16, as also confirmed by total protein measurements (Fig 3b). When medium supplemented with ED FBS and not preconditioned with cells was applied to the column, the total protein distribution was analogous, yet no CD63+/CD9+/CD81+ particles were detected (Fig 3c). We concluded that exosomes were present in SEC fractions # 5–8, with the highest exosome level in fraction # 5, as confirmed by the particle size distribution and transmission electron microscopy imaging (S2 Fig). These experiments showed that SEC concentrates exosomes in fraction #5 and the majority of serum proteins elute in later fractions (# 8–16), enabling recovery of “clean” exosomes that are depleted of bovine proteins.

### Mass spectrometry analysis of proteins in the SEC fractions

**Fig 4:**
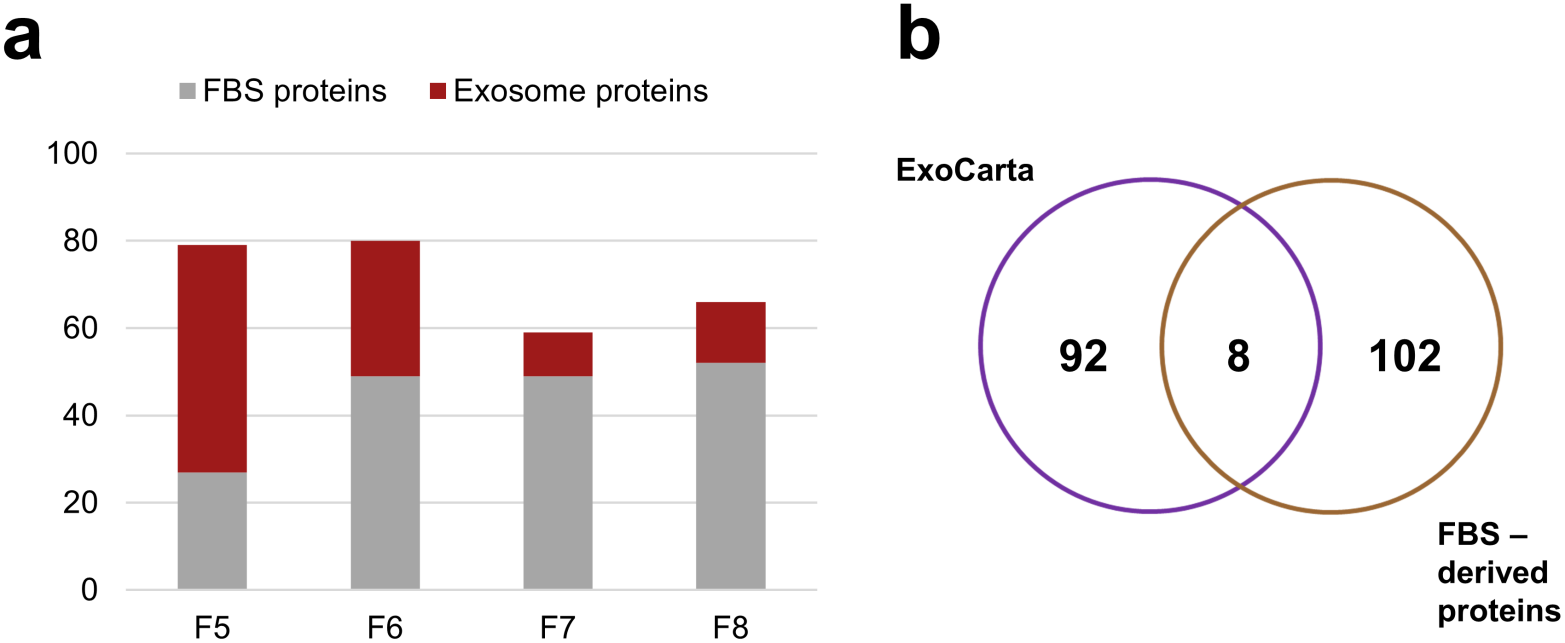
Mass spectrometry analysis of the selected SEC fractions # 5–8. In (a) the number of proteins identified by MS in each SEC fraction; the ratios of putative exosome specific (red) and FBS serum-derived proteins (gray) in each fraction are shown. In (b) the overlap between the top hundred exosomal proteins reported in the ExoCarta database and proteins detected in the “fresh” culture medium supplemented with ED FBS (SEC fractions # 5–8).

The shotgun MS analysis was applied to identify exosome proteins in SEC fractions # 5–8 of the cell culture supernatant from FaDu cells (proteins identified in each fraction are listed in the S2 Table). In a parallel experiment, a portion of medium supplemented with 5% ED FBS which was not in contact with cells (i.e., a background control sample) was processed and the same fractions # 5–8 were analyzed by MS (S2 Table). Proteins identified only in fractions of medium preconditioned with cells were considered *exosome-specific*, while proteins identified in fractions of “fresh” medium (i.e., not incubated with cells) were considered to be *serum-derived*. The results demonstrated that the highest number of exosomal proteins and the lowest number of serum proteins were detected in factions # 5 and # 6 (Fig 4a). In contrast, fractions #7 and #8 were enriched in proteins derived from FBS. The most favorable ratio of the exosome-specific to serum-derived proteins was found in fraction #5. At the same time, we noted that among proteins identified in cell-unexposed (“fresh”) medium there were a few proteins listed in ExoCarta as the most often identified exosome proteins; these included actin, thrombospondin-1, tubulin-α, heat shock protein (HSP)90-α, glyceraldehyde 3-phosphate dehydrogenase, galectin-3-binding protein, α-2-microglobulin and albumin (Fig 4b). This mistaken identification of putative FBS-derived proteins as exosome components indicates that “contamination” of exosomes with serum proteins is a common problem in the field.

### Extraction of exosome proteins for mass spectrometry

**Fig 5:**
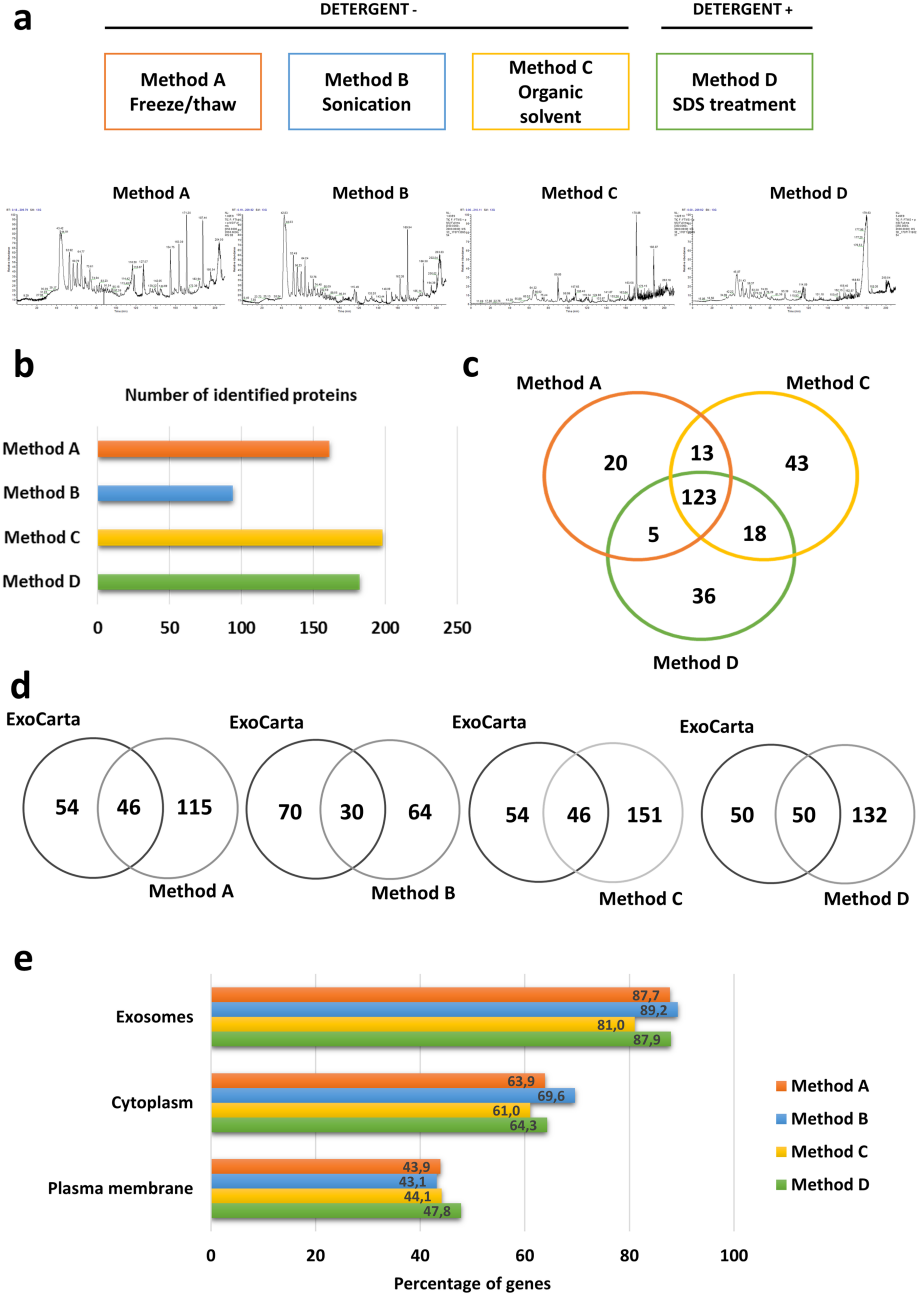
A comparison of four different methods for exosome protein extraction. In (a) the LC profiles of exosome samples processed by each of the four methods. In (b) numbers of exosome proteins identified by MS in each sample. In (c) overlapping and distinct proteins identified using Methods A, C and D. In (d) numbers of exosome proteins overlapping with or distinct from the ExoCarta database. In (e) functional enrichment analysis showing the percentages of the detected proteins that were exosome-related in each method.

Four methods commonly used for the extraction of exosome proteins were subsequently compared (Fig 5). Three methods (A, B and C) are based on detergent-free membrane disruption; method A (freeze/thaw cycles) was used to generate data presented in the previous paragraph). The fourth one (D) utilizes high concentrations of SDS to solubilize exosome membranes and is followed by *in-solution* protein digestion on the surface of a filter (filter-aided sample preparation, FASP), which allows for removal of components interfering with MS measurements. The same sample of exosomes corresponding to SEC fraction # 5 (containing about 400ng of protein) was divided into four equal portions and processed in parallel using the abovementioned methods. Surprisingly, qualitative and quantitative differences in the exosome content were visible already on the LC chromatograms of tryptic digests (Fig 5a) and were confirmed by the final results of the MS analysis. The list of proteins identified in each (sub)sample is presented in S3 Table. Significant differences in the number of identified proteins were noted in samples processed by different methods. Sonication (Method B) was found to be the least effective extraction method based on the number of detected proteins, whereas acetonitrile treatment (Method C) was the most effective, allowing for detection of nearly 200 proteins (Fig 5b). Comparisons of samples A, C and D showed that 123 proteins were present in all three samples and 99 were “method-specific” proteins (Fig 5c). The Venn diagrams in Fig 5d indicate that similar subsets of proteins detected in A, B, C and D samples overlapped with the ExoCarta list of 100 most commonly reported exosome proteins. About 50% of proteins listed in the ExoCarta were detected in each sample (S4 Table). The functional enrichment analysis (Fig 5e) using the FunRich V3 database showed that in each sample over 80% of proteins were exosome-related. Hence, there was no clear evidence that any of the four methods favored detection of exosome-related proteins. However, the presented results demonstrate that the final list of identified exosome proteins is strictly dependent on the method of protein extraction performed during sample preparation for MS.

## Discussion

The emerging role of exosomes in intercellular communication and their potential involvement in human health and disease has led to rapid development of methods for exosome isolation and characterization. MS has been extensively used for protein profiling of exosomes [11,12]. Numerous reports in the literature have provided lists of exosome proteins which are contained in the ExoCarta database [9]. In most cases, these proteins were identified by MS performed with exosomes obtained from different sources and isolated by various methods. However, while immune-based methods, such as Western blots, allow for the detection in exosomes of very low-abundant protein species such as cytokines, MS often fails to detect them. This may be because high-abundant proteins co-isolated with exosomes can mask low-abundant exosome proteins, limiting their detection and identification. Furthermore, “contaminating” plasma or serum proteins create a background noise or are taken as *bona fide* exosome components. The fact that protein species known to be serum components are present in exosome protein databases supports this conclusion. Albumin, an abundant serum component, was originally classified by Lötvall et al. [13] as an extracellular protein associated with and co-isolating with exosomes. Others considered it as a non-specific serum-derived “contaminant” [14]. In exosomes derived from serum or plasma, albumin is commonly used as a negative “purity” marker [15,16]. Another example is β-actin, a protein reported by some researchers to be enriched in exosomes [2,17], while others do not confirm its enrichment [14] or even use it as a negative “purity” marker for exosomes [18,19]. Including such controversial proteins in ExoCarta might be questionable and might be a result of many potential pitfalls in exosome harvest, isolation, and processing in preparation for MS.

Most of the data on tumor-derived exosomes resulted from studies with cell lines cultured in the presence of FBS. While the use of exosome-depleted FBS for the culture of these cell lines is commonly practiced, most investigators may not be aware that ultracentrifugation at 100,000 × g for 24 h performed in their own laboratories carries the risk of only partial removal of exosomes from FBS, leaving behind exosomes carrying bovine proteins. This is a serious problem since proteins in FBS have more than 95% of sequence homology with human serum proteins and cannot be differentiated by bioinformatics filtration. Our comparative analysis of amino acid sequences of human and bovine serum proteins that are present on the list of 100 most commonly recorded exosome proteins in ExoCarta detected high level of similarity or, in some cases, identity. Such conserved proteins are unlikely to be distinguished by MS as human or bovine and are likely to be included in the final protein score, creating an artifact. Nevertheless, processing of cell-unexposed medium as a control sample and identification of serum-derived components before establishing a final list of proteins specific for cell-derived exosome is highly recommended.

Isolation of exosomes from supernatants or plasma using SEC columns has been described as a favorable method for obtaining “clean” exosomes [20–22]. SEC is now recognized as the best method for decreasing background noise, serving as an extra purification step that allows for separation of exosomes from most, but not all, serum proteins. Our data confirm exosome enrichment in the early SEC fractions. Nevertheless, even these exosome-enriched fractions contained non-exosome proteins, which although significantly reduced in their abundance were not eliminated.

It appears that the method selected for protein extraction from isolated exosomes is critical for reliable MS analysis. The same exosome preparation extracted by four different methods, including extraction with SDS, gave quantitatively and qualitatively distinct protein profiles. Based on the number of identifications, sonication proved to be the least effective method of protein extraction, while acetonitrile treatment seemed to be the most effective. Comparisons of samples extracted using different methods revealed a similar number of proteins (with exception of the least effective sonication), yet different subsets of exosome proteins were detected in each sample originating from the same source. Therefore, the choice of the protein extraction method is a crucial decision, which can seriously affect the final results. However, a comparison of different methods of proteins extraction before initiation of a project focused on a specific subset of exosome components could be recommended.

**Fig 6:**
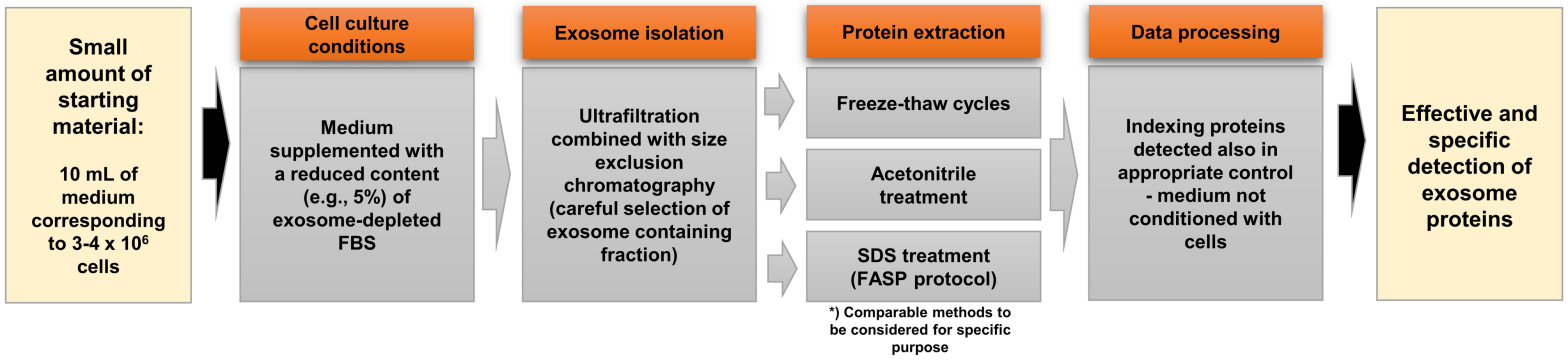
Practical hints for effective proteomics analysis of exosomes.

Based on the results reported in this study, the following recommendations for exosome harvesting, isolation, and solubilization for MS analyses should be considered (summarized in Fig 6): (a) culture media used for exosome harvesting should be supplemented with exosome-depleted FBS. However in-house depletion by ultracentrifugation is unreliable, as it does not remove all bovine contaminants. The use of ED commercial FBS is preferred as a low protein level in commercial ED FBS translates into lower background in MS; (b) bovine serum proteins may interfere with MS, accounting not only for a high background but also for potential artefacts due to high similarity in protein sequences between human and bovine proteins; (c) SEC provides an extra purification step that greatly supports MS through the separation of exosomes that elute in early fractions and before serum proteins present in later fractions. If the ratio of “exosome-specific”/"contaminating” proteins is kept low, as in early SEC fractions, the detection of low-abundance exosome proteins is facilitated; (d) the methods selected for exosome protein extraction, whether based on freeze/thaw cycles, acetonitrile treatment or SDS solubilization, are equally suitable for MS studies of low-abundance exosome proteins. However, exosome sonication is not recommended, as it yields fewer proteins than the other three procedures. These recommendations, if stringently applied to all samples, are expected to improve MS-based proteomic analyses of exosomes originating from cell cultures or body fluids. MS remains an irreplaceable tool in proteomic studies of exosomes, and the correct sample processing eliminating methodological hurdles will certainly increase the quality of MS results.

## Materials and methods

### Cell culture

All cell lines except UM-SCC6 were purchased from ATCC as the Head and Neck Cancer Panel (TCP-1012). FaDu (HTB-43) and Detroit562 (CCL-138) cells were cultured in MEM with Earle’s Salts (Sigma-Aldrich, 51412C) supplemented to the final concentration of 1 mM Sodium Pyruvate (HyClone, SH30239.01), 2 mM L-glutamine (Biowest, X0550) and 1X MEM non-essential amino acid solution (Sigma-Aldrich, M7145). SCC-9 (CRL-1629), SCC-15 (CRL-1623), SCC-25 (CRL-1628) were cultured in DMEM/F12 (HyClone, SH30023.02) supplemented with Hydrocortisone 50 μg/mL (Sigma-Aldrich, H0135). A-253 (HTB-41) cells were cultured in McCoy’s 5A (HyClone, SH30200.02). The UM-SCC6 cells were authenticated with STR-based method and then cultured in DMEM (Sigma-Aldrich, D6429) supplemented with 1x MEM non-essential amino acid solution. The final concentration of non-heat inactivated FBS (HyClone, SH30088.03) for all culture media was 10% (v/v) with gentamicin at 0.16 mg/mL. The medium was replaced 3 times per week, and cells were incubated at 37°C, in the air with 5% CO_2_. Cells between passages 8–16 were harvested. For comparative studies and for exosome isolation, cultures were supplemented with non-heat inactivated exosome-depleted (ED) FBS (Gibco, A2720801).

### Cell viability

Cell viability was assessed using CellTiter 96 Aqueous One Solution Reagent (Promega, G3582). The absorbance of colored formazan, a bioconversion product of 3-(4,5-dimethylthiazol-2-yl)-5-(3-carboxymethoxyphenyl)-2-(4-sulfophenyl)-2H-tetrazolium was measured at 490 nm using a 96-well plate reader (BioTek, Epoch). Cell suspensions containing 5 × 103–1 × 10^4^ cells/100 μL were plated in wells of a 96-well cell culture plate (Greiner Bio-One, 655180) and incubated for 48h. Then, the culture medium was replaced with fresh medium supplemented with a pre-defined dose of standard (STD) FBS (HyClone) or exosome-depleted (ED) FBS (Gibco). Cells were cultured for 24 h or 48 h. Next, the medium was removed and replaced with the MTS substrate in serum-free medium containing phenol red. Measurements were made every 20–30 min during a 3 h incubation in 5% CO_2_ in air at 37°C.

### Acetylcholinesterase activity assay

Acetylcholinesterase activity was measured in each ultracentrifugation-derived fraction diluted 5x with PBS. A 50 μL aliquot of each sample was added to wells of a 96-well flat-bottom microplate (Greiner Bio-One, 655101). Serial dilutions of acetylcholinesterase (0 to 300 mU/mL) were used as standards. Immediately before measurement, a 50 μL aliquot of the reaction mixture (0.2 mM 5,5′-dithiobis(2-nitrobenzoic acid) and 2.5 mM acetylthiocholine) was added to each well, and the change in absorbance at 412 nm was measured after 20 min incubation.

### Electrophoresis and protein immunodetection

Proteins were mixed with a loading buffer (12% SDS, 0.6% bromophenol blue, 60% glycerol, and optionally 600 mM DTT) at the v/v ratio of 1:5 and denatured for 5min at 95°C. Separation was performed using 11% home-made polyacrylamide gels. After the sample transfer (100 V, 60 min), nitrocellulose membranes (Thermo Scientific, 88018) were blocked for 1 h at RT (5% non-fat dry milk in 150 mM NaCl, 10 mM Tris pH 7.5, 0.1% Tween-20) and incubated overnight with a primary antibody (anti-CD63: Invitrogen, 10628D, 1:1500; anti-CD81: Invitrogen, 10630D, 1:800; anti-CD9: Invitrogen, 10626D, 1:800). After a triple wash (150mM NaCl, 10mM TRIS pH 7.5, 0.1% Tween-20) a secondary HRP-conjugated antibody (Themo Scientific, 31430) was added for 1 h of incubation. Bands were detected using SuperSignal West Femto Maximum Sensitivity Substrate (Thermo Scientific, 34095) diluted at the volume ratio of 1:10 with washing buffer. CD63 and CD81 were detected under non-reducing conditions. Visualization of proteins on nitrocellulose membranes or in polyacrylamide gels was performed using Ponceau S reversible staining (Sigma, P7170) or PageBlue Protein Staining Solution (Thermo Scientific, 24620), respectively. A Perfect Tricolor Protein Ladder (Eurx, E3210-01) was used as a protein molecular weight marker.

### Exosome isolation

Cells were cultured in T175 flasks (Greiner BioOne, 660175). 24 h before exosome harvest, cell culture medium was replaced with fresh medium containing 5% (v/v) ED FBS. A day later, cell culture medium containing released exosomes was collected, pre-cleared from the remaining cells or cellular debris by a series of centrifugations at 200, 2000 and 10,000 × g for 2 × 10 and 30 min, respectively. The supernatant was filtered using a 0.22 μm syringe filter unit (Roth, PA49.1). Exosome enrichment was achieved using a Vivacell 100 centrifugal device (100 kDa molecular weight cut-off; Sartorius, VC1042). An exosome sample (1 mL) was loaded onto ready-to-use size exclusion chromatography columns (Izon Science). Fractions (1 mL) were eluted with PBS without divalent cations. The first fraction was collected right after the sample had been loaded and the total of 24 mL was collected. Samples were stored at -80°C until use.

### Preparation of exosome-depleted cell culture medium

Cell culture medium was prepared according to the protocol of Théry et al. [10]. Briefly, medium complemented with all required nutrients and 20% (v/v) FBS was ultracentrifuged overnight at 100,000 × g at 4°C (SW28 Ti swinging-bucket rotor, Beckman Coulter). Then, the collected supernatant was sterilized with the use of a 0.22-μm syringe filter and stored at 4°C. The desired concentration was obtained by diluting the complete medium without FBS.

### Exosome proteins extraction and sample preparation for mass spectrometry analysis

Exosomes were extracted using four different methods. (A) freeze-thaw cycles; (B) sonication; (C) acetonitrile treatment; (D) SDS treatment. For comparison of protein extraction efficiency, the SEC fraction #5 (containing approximately 0.4 μg of protein) was divided into four equal aliquots and processed as follows. Sample A was subjected to 10 freeze-and-thaw cycles, each lasting for 1 min in liquid nitrogen and 3 min at 95°C. Sample B was sonicated using Bioruptor Plus for 20 cycles [30 sec ON/30 sec OFF]. Sample C was mixed with acetonitrile to the final concentration of 50% (v/v), and after 45 min of incubation at RT with occasional vortexing, acetonitrile was evaporated using a centrifugal vacuum concentrator. Sample D was mixed with a lysis buffer (4% SDS, 100mM Tris/HCl pH 7.6, 0.1M DTT) in the volume ratio of 1:1 and incubated at 95°C for 5 min. Subsequently, samples A-C were further processed using a standard *in solution digestion* protocol. Briefly, 250 mM solution of ammonium bicarbonate was added to each sample to reach the final concentration of 25 mM. The mixture was reduced in the presence of 5 mM DTT for 15 min at 60°C. After cooling to RT, alkylation was performed by incubation with iodoacetamide (15 mM) in darkness for 30 min. An extra portion of the reducing agent was added for an additional 15 min of incubation to avoid trypsin alkylation by the leftover alkylation agent. Next, all samples were treated with modified trypsin (Promega, V5111) at the final concentration of 4 ng/μL per sample. Sample D was processed according to a standard FASP protocol [23] with minor modifications. The denatured and reduced protein extract was mixed with 8 M urea (Sigma, U5128) in 0.1 M Tris/HCl pH 8.5 in the volume ratio of 1:4 ratio, loaded onto a Microcon YM-10 (Millipore, Cat. MRCPRT010) filtration device and centrifuged for 20 min. After additional washing with the urea solution, a 50 μL aliquot of 50 mM iodoacetamide solution in 8 M urea solution was added, mixed at 600 rpm in a heat block for 1 min and incubated without mixing for 20 min at RT. Three steps of washing with 100 μL of urea solution were performed before double rinsing with 150 μl of a digestion buffer (25mM ammonium bicarbonate), each time followed with centrifugation at 14000 × g for 25 min. Then, 40 μL of trypsin in the digestion buffer was added onto the filter membrane and mixed at 600 rpm for 1 min. All samples were digested in a *humid chamber* at 37°C for 18h. Next day, sample D was centrifuged at 14,000 × g for 25 min to recover peptides after digestion. Samples A-C, as well as the filtrate of sample D, were then stored at -80°C until mass spectrometry analysis.

### Mass spectrometry analysis

Identification and quantitation of exosome proteins were performed using the Dionex UltiMate 3000 RSLC nanoLC System connected to QExactive Orbitrap mass spectrometer (Thermo Fisher Scientific). Peptides derived from in-solution digestion were separated on a reverse phase Acclaim PepMap RSLC nanoViper C18 column (75 μm × 25 cm, 2 μm granulation) using acetonitrile gradient (from 4 to 60%, in 0.1% formic acid) at 30°C and a flow rate of 300 nL/min (for 230 min). The spectrometer was operated in data-dependent MS/MS mode with survey scans acquired at a resolution of 70,000 at m/z 200 in MS mode, and 17,500 at m/z 200 in MS2 mode. Spectra were recorded in the scanning range of 300–2000 m/z in the positive ion mode. Higher energy collisional dissociation (HCD) ion fragmentation was performed with normalized collision energies set to 25.

For proteins identification and quantitation, obtained raw files were analyzed by Proteome Discoverer (PD), version 1.4.14 (Thermo Fisher Scientific). The identification of proteins by PD was performed using both Sequest and the Mascot engines against the UniProt Complete Proteome Set of Humans (123,619 sequences; the database access in March 2017) using the following parameters identical for both search engines: a tolerance level of 10 ppm for MS and 0.08 Da for MS/MS. Trypsin was used as the digesting enzyme, and two missed cleavages were allowed. The carbamidomethylation of cysteines was set as a fixed modification, and the oxidation of methionines was allowed as a variable modification. Identifications were validated using Percolator node based on q-value with FDR 0.01 for both proteins and peptides. To perform protein quantitation, Precursor Ion Area Detector node was used, followed by data export to Excel where normalization based on total ion current was done.

### Statistical analysis

Functional-enrichment analysis for GO-terms was performed using FunRich v3.0 software [24]. For annotation of identified proteins, the FunRich database was used. Enriched terms in cellular components category were assessed by hypergeometric test and ranked by *p*-value using FunRich. A *p* < 0.05 was considered significant. The UniProt identifiers were converted to gene name using FunRich and UniProt Retrieve/ID mapping tools. The gene names were used as an input for presented analyses of mass spectrometry data.

## Supporting information

S1 Fig A Venn diagram showing the overlap between proteins identified when mass spectrometry data from exosome studies were used to search for overlapping human and bovine protein sequences. (PDF)

S2 Fig Characteristics of exosomes. (a) The size distribution profile of exosomes was determined by a Zetasizer Nano-ZS90 instrument (Malvern Instruments). Samples (60μL) were analyzed immediately after isolation at the constant temperature (20°C) with dedicated disposable low-volume cuvettes (ZEN0118, Malvern). Data were acquired and analyzed using Malvern Zetasizer Software 7.12. The dispersant refractive index was 1.330 (ICN PBS Tablets) and equilibration time was set for 30 s. In single analysis 10 measurements with 10 runs in automatic mode were performed and averaged. The results are displayed as particle size distribution by number. Presented graph shows results for fraction #5. (b) For visualization of vesicles a 5 μL of exosomes suspension was loaded on a collodion-carbon coated copper grid (300 mesh). Negative staining was performed with 1% aqueous uranyl acetate. The air dried grids were analyzed using transmission electron microscopy Tecnai G2 T12 Spirit BioTwin FEI. TEM analysis was performed by Laboratory of Electron Microscopy, Faculty of Biology, University of Gdansk. Presented images show exosomes in fraction #5.
(PDF)

S1 Table List of 20 most commonly reported exosomal proteins according to ExoCarta database: The similarity between human and bovine amino acid sequences. (XLSX)

S2 Table List of proteins identified in individual fractions 5–8 of SEC separation. (XLSX)

S3 Table Exosome proteins identified depending on extraction method. (XLSX)

S4 Table The presence of exosome proteins listed in the ExoCarta in sample extracted by four different methods. (XLSX)
