# Supplementary material for: Harmonization of exosome isolation from culture supernatants for optimized proteomics analysis
Source: PLoS One. 2018 Oct 31;13(10):e0205496. doi: 10.1371/journal.pone.0205496 (PMC6209201; doi:10.1371/journal.pone.0205496)
Supplement: S1 Fig — (PDF) [file pone.0205496.s001.pdf]

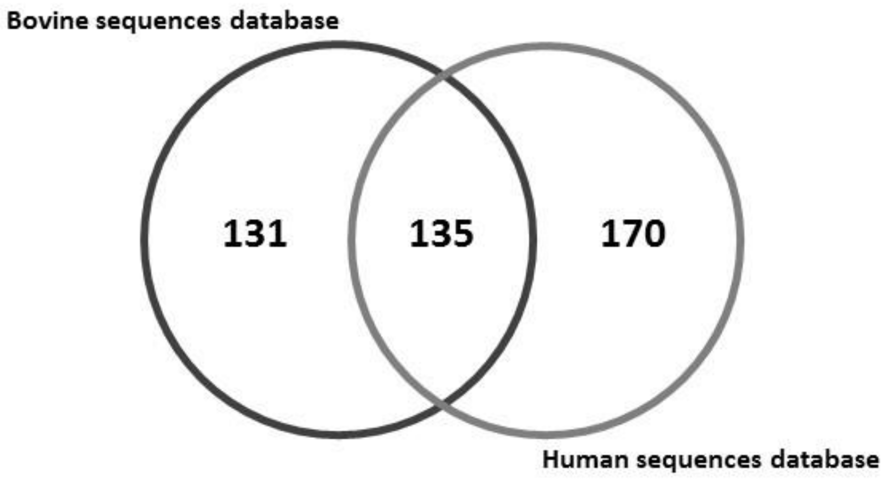

Gene names of overlapping proteins														
A2M	ANXA11	ARF6	CD82	COL6A3	F2	GNAS	HSP90AA1	ITIH2	KRT4	MFGE8	RAB10	RRAS2	SLC3A2	TUBB1
ACAN	ANXA2	ARRDC1	CD9	CPNE8	F5	GNB1	HSP90AB1	ITIH3	KRT5	MSN	RAB11A	S100A10	SLC44A1	TUBB4B
ACTB	ANXA5	ATP1A1	CFL1	CTNNA1	FBLN1	GNB2	HSPA8	JUP	KRT77	NEB	RAB1B	S100A14	SLC44A2	VCAN
ACTG2	ANXA7	BSG	CHMP5	CTNND1	FGB	GP1BB	IST1	KRT1	LAMA5	NID1	RAB5C	SDC1	SNPH	VPS4B
ADAM10	APOA1	C3	CLDN4	EEF1A1	FGG	GPC1	ITGA2B	KRT10	LAMB2	NRAS	RAB7A	SDCBP	STOM	VTA1
AGRN	APOB	C9	CLTC	EGFR	FLOT2	GSTP1	ITGA3	KRT14	LAMC1	PDCD6	RAP1A	SERPINC1	THBS1	VTN
AHSG	APOC3	CAV1	COL18A1	EPCAM	FN1	H2AFZ	ITGA6	KRT17	LAMP2	PDCD6IP	RAP1B	SLC1A5	TSG101	VWF
ALB	APOE	CD151	COL1A1	EPHA2	GAPDH	H3F3C	ITGB1	KRT18	LGALS3BP	PPIA	RAP2B	SLC2A1	TSPAN14	YWHAE
ANXA1	APOM	CD81	COL6A1	F13A1	GNAI2	HAPLN1	ITGB3	KRT2	MAMLD1	PTGFRN	RPL18	SLC2A3	TUBA4A	YWHAZ
