# Supplementary figures and images for: Harmonization of exosome isolation from culture supernatants for optimized proteomics analysis
Source: PLoS One. 2018 Oct 31;13(10):e0205496. doi: 10.1371/journal.pone.0205496 (PMC6209201; doi:10.1371/journal.pone.0205496)

**a**

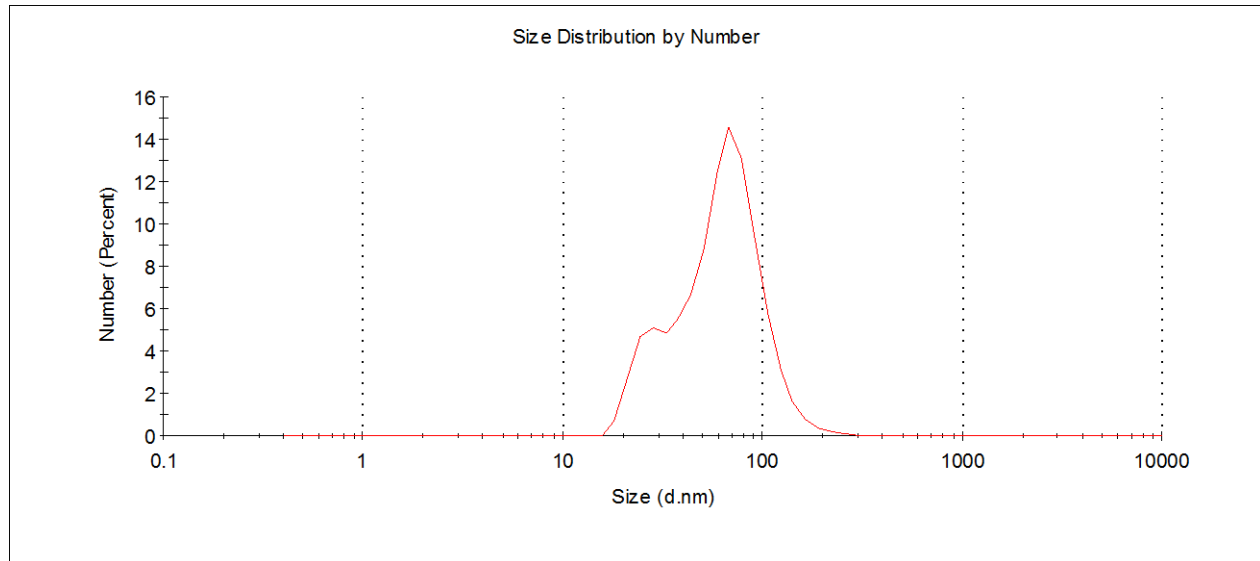

**b**

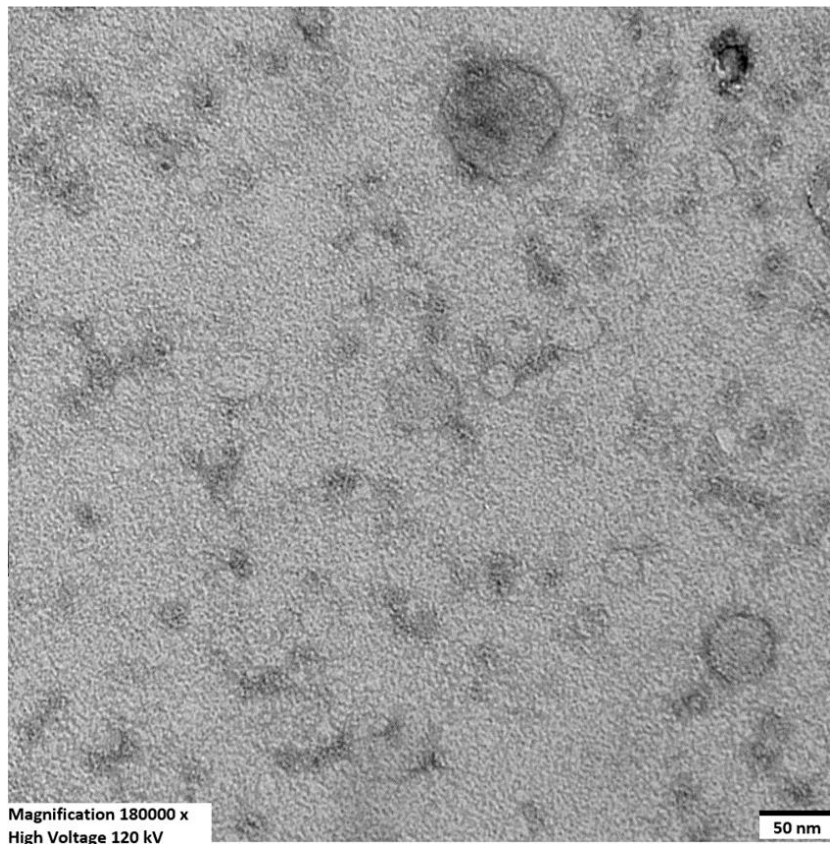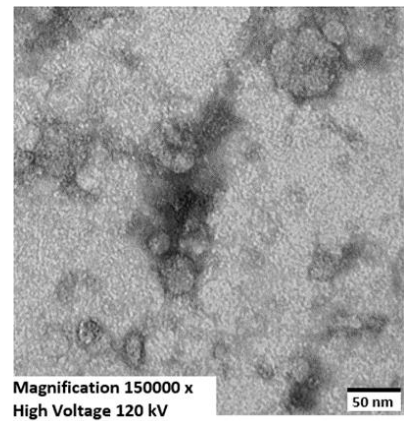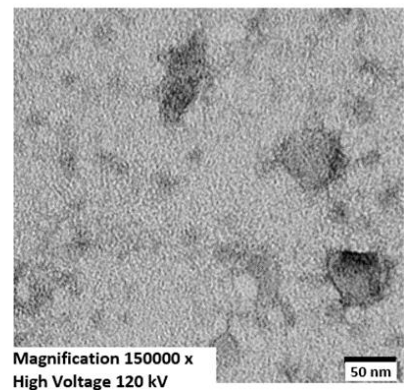

Supplement: S2 Fig — (a) The size distribution profile of exosomes was determined by a Zetasizer Nano-ZS90 instrument (Malvern Instruments). Samples (60μL) were analyzed immediately after isolation at the constant temperature (20°C) with dedicated disposable low-volume cuvettes (ZEN0118, Malvern). Data were acquired and analyzed using Malvern Zetasizer Software 7.12. The dispersant refractive index was 1.330 (ICN PBS Tablets) and equilibration time was set for 30 s. In single analysis 10 measurements with 10 runs in automatic mode were performed and averaged. The results are displayed as particle size distribution by number. Presented graph shows results for fraction #5. (b) For visualization of vesicles a 5 μL of exosomes suspension was loaded on a collodion-carbon coated copper grid (300 mesh). Negative staining was performed with 1% aqueous uranyl acetate. The air dried grids were analyzed using transmission electron microscopy Tecnai G2 T12 Spirit BioTwin FEI. TEM analysis was performed by Laboratory of Electron Microscopy, Faculty of Biology, University of Gdansk. Presented images show exosomes in fraction #5. (PDF) [file pone.0205496.s002.pdf]
